# Clinical impact of very early recurrence after conversion surgery for stage IV gastric cancer

**DOI:** 10.1002/ags3.12738

**Published:** 2023-09-14

**Authors:** Atsushi Morito, Kojiro Eto, Masaaki Iwatsuki, Tasuku Toihata, Keisuke Kosumi, Shiro Iwagami, Yoshifumi Baba, Yuji Miyamoto, Naoya Yoshida, Hideo Baba

**Affiliations:** ^1^ Department of Gastroenterological Surgery, Graduate School of Medical Sciences Kumamoto University Kumamoto Japan

**Keywords:** conversion surgery, prognostic nutritional index, stage IV gastric cancer, very early recurrence

## Abstract

**Background:**

The development and improved response to chemotherapy has resulted in a survival benefit of conversion surgery (CS) for advanced gastric cancer (GC). However, this benefit is limited in some cases, such as in those with very early recurrence (VER). This study investigated the relationship between outcome and clinicopathological characteristics after CS for stage IV GC, and the risk factors for VER after CS.

**Methods:**

We retrospectively studied 184 patients with stage IV GC who initially underwent chemotherapy, including 36 patients who underwent CS between May 2007 and January 2022. We evaluated the long‐term outcome after CS for stage IV GC and the clinicopathological characteristics of the patients who underwent CS.

**Results:**

Median survival times (MSTs) in the chemotherapy alone and CS groups were 13.4 and 36.5 months, respectively (*p* < 0.0001). Of the 27 patients who underwent R0 resection, 22 remained free of early recurrence and five experienced VER. MSTs in the VER and free of early recurrence groups were 15.2 and 44.1 months, respectively (*p* < 0.0001). Significantly more patients had liver metastasis before initial treatment in the VER group than in the FER group (*p* = 0.016). There were more patients with preoperative PNI <40 in the VER group (*p* = 0.046).

**Conclusion:**

CS is an effective treatment for stage IV GC, but VER is associated with poor prognosis. We need to carefully consider the indications for CS, especially for patients with poor nutritional status and liver metastases.

## Graphical abstract

This study demonstrated that conversion surgery is an effective treatment but that very early recurrence within 6 months is associated with a poor prognosis. We need to carefully consider the indication of conversion surgery, especially for the patients who have a poor nutritional status and liver metastases.

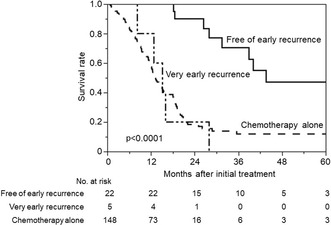

## 1 INTRODUCTION

Gastric cancer (GC) is the fifth most common cancer and fourth leading cause of cancer‐related deaths worldwide.^1^ Although early GC is curable, advanced GC is still associated with poor prognosis. The initial treatment option for patients with stage IV GC is palliative systemic chemotherapy.^2^ However, the median survival time (MST) for stage IV GC is an unsatisfactory 7–14 months.^3^, ^4^ To achieve a better treatment outcome for GC patients, it is important to improve the long‐term outcome of patients with stage IV GC.

The development of anticancer drugs and effective regimens has resulted in remarkable tumor shrinkage and disappearance of distant metastasis, and some stage IV GC patients have been able to undergo curative resection. Curative resection following systemic chemotherapy in initially unresectable GC is called conversion surgery (CS), and a survival benefit has been reported.^5^, ^6^, ^7^

Some patients experience very early recurrence (VER) after curative surgery. VER refers to recurrence within 6 months after resection and has been reported in patients with GC, hepatocellular carcinoma, intrahepatic cholangiocarcinoma, and colorectal liver metastases. Patients with VER have poor prognosis.^8^, ^9^, ^10^, ^11^, ^12^ In particular, post‐gastrectomy patients are often unable to receive adequate chemotherapy because of postoperative complications and poor nutrition from decreased oral intake, increased toxicity, and decreased tolerability of chemotherapy.^13^ In such cases, the prognosis may be better with continued chemotherapy without CS.

The purpose of this study was to evaluate the relationship between the outcome and clinicopathological characteristics after CS for stage IV GC, and discuss the factors involved in VER after CS.

## 2 PATIENTS AND METHODS

### 2.1 Patients

We retrospectively analyzed data from 184 patients diagnosed with unresectable stage IV GC who underwent chemotherapy as the initial treatment, including 36 who underwent surgery with curative intent, between May 2007 and January 2022. The clinicopathological data were retrospectively reviewed in accordance with the Japanese Classification of Gastric Carcinoma.^14^ Initially, all patients were pathologically diagnosed with gastric adenocarcinoma with distant metastatic lesions, including liver, lung, peritoneal, para‐aortic lymph node, and other distant metastases. We excluded patients with recurrent GC, patients with peritoneal lavage cytology positive disease without any other metastatic sites, patients receiving less than one cycle of chemotherapy, and patients whose response to chemotherapy could not be assessed.

### 2.2 Treatments

First‐line chemotherapy was cisplatin, oxaliplatin, fluorouracil, S‐1, or paclitaxel, which was decided by clinicians. Trastuzumab was administered for human epidermal growth factor receptor‐2‐positive patients. Some patients received chemotherapy with immune checkpoint inhibitor in clinical trials. The response to chemotherapy was assessed after each set of 2–4 cycles and was classified according to the Response Evaluation Criteria in Solid Tumors.^15^ When GC that was initially regarded as unresectable responded well to chemotherapy, gastrectomy and/or metastasectomy was considered if R0 resection were possible. Proximal, distal, or total gastrectomy was selected according to tumor location and size. Technically resectable metastases, such as in number 16 lymph nodes, liver, ovaries, pancreas, or transverse colon, which arose by direct invasion from the primary tumor, were removed simultaneously.

### 2.3 Evaluations

The prognostic nutritional index (PNI) was used for preoperative assessment of immune and nutritional status. Blood samples and laboratory measurements collected up to 1 week prior to surgery were considered for inclusion in the PNI assessment. PNI was determined as follows: (10 × serum albumin [g/dL]) + (0.005 × total lymphocyte count). We used PNI <40 as the cutoff value according to the result of receiver operating characteristic curve analysis. Postoperative complications were graded according to the Clavien–Dindo classification.^16^ Pathological response was evaluated according to the Japanese Classification of Gastric Carcinoma^14^: grade 0, no part of the tumor affected; grade 1a, less than one‐third affected; grade 1b, between one‐third and two‐thirds affected; grade 2, more than two‐thirds affected; and grade 3, no residual tumor. Pathological response was defined as one‐third or more of the tumor affected (grade 1b, 2, or 3). Overall survival (OS) was measured from the date of chemotherapy initiation until death from GC or other causes.

### 2.4 Follow‐up

Postoperative adjuvant chemotherapy until recurrence consisted of S‐1 monotherapy or S‐1 combined with another drug, depending on the results of the postoperative pathological examination, performance status and nutritional status. All patients underwent computed tomography at least every 3 months during the first 3 years, followed by every 6 months until 5 years after surgery. Recurrence within 6 months after gastrectomy was defined as VER.

### 2.5 Statistical analysis

The clinicopathological characteristics and laboratory data of the two groups were compared using the χ^2^ test for categorical variables and Mann–Whitney *U* test for continuous variables. *p* < 0.05 was considered statistically significant. The sensitivity and specificity of the nominated variables to predict VER were assessed using receiver operating characteristic curve analysis. Goodness‐of‐fit was assessed by calculating the area under the curve (AUC), and the optimal cut‐off value was determined using the Youden index. OS was estimated using the Kaplan–Meier method, and OS differences were compared using the log‐rank test. All tests were analyzed using JMP version 16.0 (SAS Institute).

## 3 RESULTS

### 3.1 Comparison of long‐term outcomes between chemotherapy alone and CS groups

**FIGURE 1:**
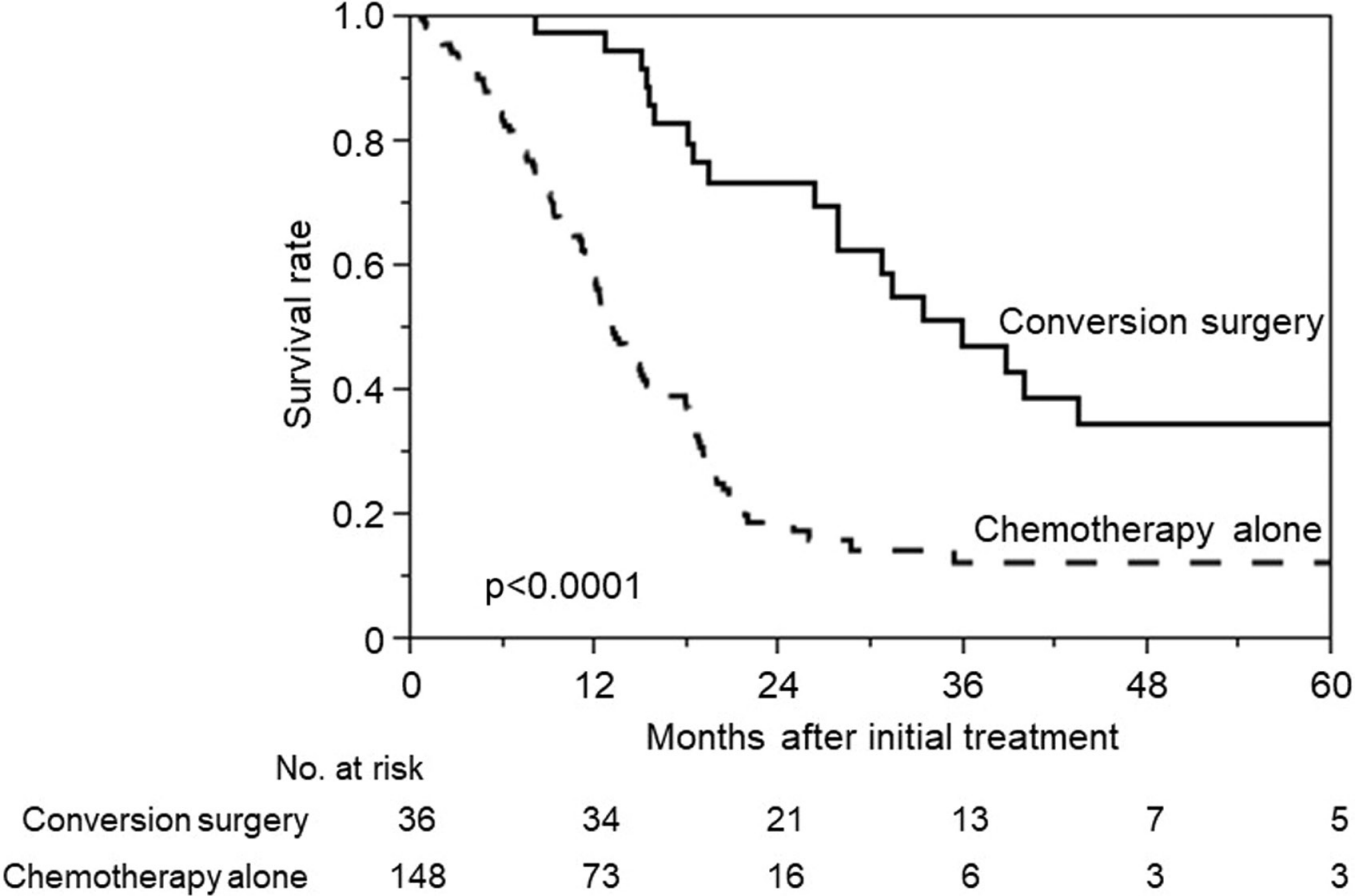
Kaplan–Meier analysis of overall survival in the conversion surgery and chemotherapy alone groups after initial treatment.

MSTs in the chemotherapy alone and CS groups were 13.4 and 36.5 months, respectively (Figure 1). The CS group had significantly better survival after initial treatment (*p* < 0.0001).

### 3.2 Characteristics of patients who underwent CS

Thirty‐six patients underwent CS with curative intent (Table 1). There were 23 men and 13 women, with a median age of 61 years. Before initial treatment, 18 patients (50%) had distant lymph node metastases, seven (19%) had liver metastases, 20 (56%) had peritoneal metastases, and eight (22%) had positive peritoneal lavage cytology. Twenty patients had two or more noncurative factors. Histologically, 16 patients had intestinal type and 20 had diffuse type tumors. Seven patients received molecularly targeted therapy and four received immune checkpoint inhibitors.

**TABLE 1:** Characteristics and chemotherapy results of 36 patients who underwent conversion surgery.

Factors	Variable	*N* = 36
Age, year	Median (range)	61 (35–77)
Sex, *n*	Male/female	23/13
Body mass index, kg/m^2^	Median (range)	22.1 (15.7–27.9)
ASA‐PS, *n*	1/2/3	1/32/3
Histology, *n*	Intestinal/diffuse	16/20
Tumor location, *n*	U/M/L/whole	9/6/5/16
cT stage, *n* ^a^	2/3/4	1/4/31
cN stage, *n* ^a^	1/2/3	7/11/18
Distant metastases		
Lymph nodes, *n* (%)	Present	18 (50)
Liver, *n* (%)	Present	7 (19)
Peritoneum, *n* (%)	Present	20 (56)
Ovary, *n* (%)	Present	2 (6)
Peritoneal lavage cytology, *n* (%)	Positive	8 (22)
Noncurative factor, *n*	1/≥2	16/20
First‐line chemotherapy		
Base‐line chemotherapy, *n*	Platinum/taxane	32/4
Molecularly targeted, *n* (%)	Present	7 (19)
Immune checkpoint inhibitor, *n* (%)	Present	4 (11)
No. of courses, *n*	<5/≥5	15/21
CEA, *n*	≤5/>5	32/4
CA19‐9, *n*	≤37/>37	31/5
Preoperative PNI, *n*	<40/≥40	6/30

Abbreviations: ASA‐PS, American Society of Anesthesiologists‐Physical Status; CA19‐9, carbohydrate antigen, 19–9; CEA, carcinoembryonic antigen; PNI, prognostic nutritional index.

^a^ According to the Japanese Classification of Gastric Carcinoma (3rd English edition).

### 3.3 Surgical and pathological results in patients who underwent CS

R0 resection was performed in 27 patients who underwent CS, R1 resection in five, and R2 resection in four (Table 2). Four patients resulted in R1 resection due to positive peritoneal lavage cytology, and one patient had positive resection margins. All cases that resulted in R2 resection were due to peritoneal dissemination. Twenty‐eight patients underwent total gastrectomy. The median operating time was 358 min and median intraoperative blood loss was 558 mL. Grade 1a, 1b, 2, and 3 pathological response of the primary tumor was seen in 11, 11, 12, and two patients, respectively. Severe postoperative complications of Clavien–Dindo grade 3a or higher were observed in nine patients (25%).

**TABLE 2:** Surgical and pathological findings of 36 patients who underwent conversion surgery.

Factors	Variable	*N* = 36
Operative procedure	DG/TG/PG	6/28/2
Combined resection		
Liver, *n* (%)	Present	5 (14)
Pancreas, *n* (%)	Present	3 (8)
Colon, *n* (%)	Present	3 (8)
Spleen, *n* (%)	Present	4 (11)
Para‐aortic lymph node dissection, *n* (%)	Present	5 (14)
Ovary, *n* (%)	Present	2 (6)
Operating time, min	Median (range)	358 (187–677)
Blood loss, mL	Median (range)	558 (11–2460)
Residual tumor, *n*	R0/R1/R2	27/5/4
ypT stage, *n* ^a^	0/1/2/3/4	2/2/7/10/15
ypN stage, *n* ^a^	0/1/2/3	16/6/7/7
Pathological response, *n*	1a/1b/2/3	11/11/12/2
Postoperative complications		
Pancreatic fistula, *n* (%)	Present	5 (14)
Leakage, *n* (%)	Present	3 (8)
Abdominal abscess, *n* (%)	Present	3 (8)
CDc≥2, *n* (%)	Present	12 (33)
CDc≥3, *n* (%)	Present	9 (25)
Postoperative chemotherapy, *n* (%)	Present	31 (86)
Very early recurrence, *n* (%)	Present	5 (14)

^a^ According to the Japanese Classification of Gastric Carcinoma (3rd English edition).

Abbreviations: CDc, Clavien–Dindo classification; DG, distal gastrectomy; PG, postal gastrectomy; TG, total gastrectomy.

### 3.4 Comparisons of patient characteristics between VER and free of early recurrence groups

In 27 patients who underwent radical resection, 22 remained free of early recurrence (FER) and five patients experienced VER within 6 months after CS (Table 3). VER included three cases of liver metastasis (60%), two of peritoneal dissemination (40%), and two of distant lymph node metastasis (40%). Significantly more patients had liver metastasis before initial treatment in the VER group than in the FER group (*p* = 0.016). There were more patients with preoperative PNI <40 in the VER group (*p* = 0.046).

**TABLE 3:** Characteristics and results of chemotherapy in 27 patients who underwent radical resection by conversion surgery.

Factors	Variable	FER	VER	*p* Value
		*N* = 22	*N* = 5	
Age, year	Median (range)	63 (35–77)	57 (49–76)	0.82
Sex, *n*	Male/female	13/9	4/1	0.36
Body mass index, kg/m^2^	Median (range)	22.2 (15.7–27.9)	22.4 (17.4–24.3)	0.75
ASA‐PS, *n*	2/3	20/2	5/0	0.35
Histology, *n*	Intestinal/diffuse	10/12	2/3	0.82
Tumor location, *n*	U/M/L/whole	2/5/4/11	2/1/0/2	0.28
cT stage, *n* ^a^	2/3/4	0/3/19	1/1/3	0.15
cN stage, *n* ^a^	1/2/3	6/5/11	1/2/2	0.74
Distant metastases				
Lymph node, *n* (%)	Present	11 (50)	2 (40)	0.68
Liver, *n* (%)	Present	2 (9)	3 (60)	0.016
No. of metastases, *n*	Median (range)	3.5 (3–4)	3 (2–7)	0.76
Maximum size, mm	Median (range)	10.5 (9.6–10.5)	10 (8–20)	1.00
Peritoneum, *n* (%)	Present	14 (63)	2 (40)	0.33
Ovary, *n* (%)	Present	2 (9)	0 (0)	0.35
Peritoneal lavage cytology, *n* (%)	Positive	4 (18)	0 (0)	0.18
Noncurative factor, *n*	1/≥2	9/13	3/2	0.43
First‐line chemotherapy				
Base‐line chemotherapy, *n*	Platinum/Taxane	21/1	4/1	0.29
Molecularly targeted, *n* (%)	Present	3 (14)	1 (20)	0.72
Immune checkpoint inhibitor, *n* (%)	Present	3 (14)	0 (0)	0.25
No. of courses, *n*	<5/≥5	10/12	3/2	0.55
CEA, *n*	≤5/>5	20/2	5/0	0.35
CA19‐9, *n*	≤37/>37	21/1	4/1	0.29
Preoperative PNI, *n*	<40/≥40	1/21	2/3	0.046

^a^ According to the Japanese Classification of Gastric Carcinoma (3rd English edition).

Abbreviations: ASA‐PS American Society of Anesthesiologists‐Physical Status; CEA, carcinoembryonic antigen; CA19‐9, carbohydrate antigen 19–9; FER, free of early recurrence; PNI, prognostic nutritional index; VER, very early recurrence.

### 3.5 Comparison of surgical and pathological results between the VER and FER groups

The surgical and pathological findings of 27 patients who underwent R0 CS according to early recurrence are shown in Table 4. Compared with the FER group, the VER group experienced anastomotic leakage (*p* = 0.046) more often. The pathological response grade was lower in the VER group (*p* = 0.028).

**TABLE 4:** Surgical and pathological findings of 27 patients who underwent radical resection by conversion surgery.

Factors	Variable	FER	VER	*p* Value
		*N* = 22	*N* = 5	
Operative procedure	DG/TG/PG	5/16/1	0/4/1	0.21
Combined resection				
Liver, *n* (%)	Present	2 (9)	2 (40)	0.11
Pancreas, *n* (%)	Present	1 (5)	0 (0)	0.51
Colon, *n* (%)	Present	1 (5)	0 (0)	0.51
Spleen, *n* (%)	Present	3 (14)	0 (0)	0.25
Para‐aortic lymph node dissection, *n* (%)	Present	4 (18)	0 (0)	0.18
Ovary, *n* (%)	Present	2 (9)	0 (0)	0.35
Operating time, min	Median (range)	308 (187–658)	461 (299–538)	0.07
Blood loss, mL	Median (range)	561 (11–2460)	1525 (110–2228)	0.24
ypT stage, *n* ^a^	0/1/2/3/4	2/2/5/8/5	0/0/2/1/2	0.58
ypN stage, *n* ^a^	0/1/2/3	14/3/2/3	1/2/2/0	0.10
Pathological response, *n*	1a/1b/2/3	2/8/10/2	3/2/0/0	0.028
Postoperative complications				
Pancreatic fistula, *n* (%)	Present	2 (9)	0 (0)	0.35
Leakage, *n* (%)	Present	1 (5)	2 (40)	0.046
Abdominal abscess, *n* (%)	Present	1 (5)	0 (0)	0.51
CDc ≥ 2, *n* (%)	Present	5 (23)	2 (40)	0.44
CDc ≥ 3, *n* (%)	Present	3 (14)	2 (40)	0.20
Adjuvant chemotherapy, *n* (%)	Present	19 (86)	3 (60)	0.20

^a^ According to the Japanese classification of gastric carcinoma (3rd English edition).

Abbreviations: CDc, Clavien–Dindo classification; CS, conversion surgery; DG, distal gastrectomy; FER, free from early recurrence; PG, postal gastrectomy; TG, total gastrectomy; VER, very early recurrence.

### 3.6 Comparison of long‐term outcomes between the VER and FER groups

**FIGURE 2:**
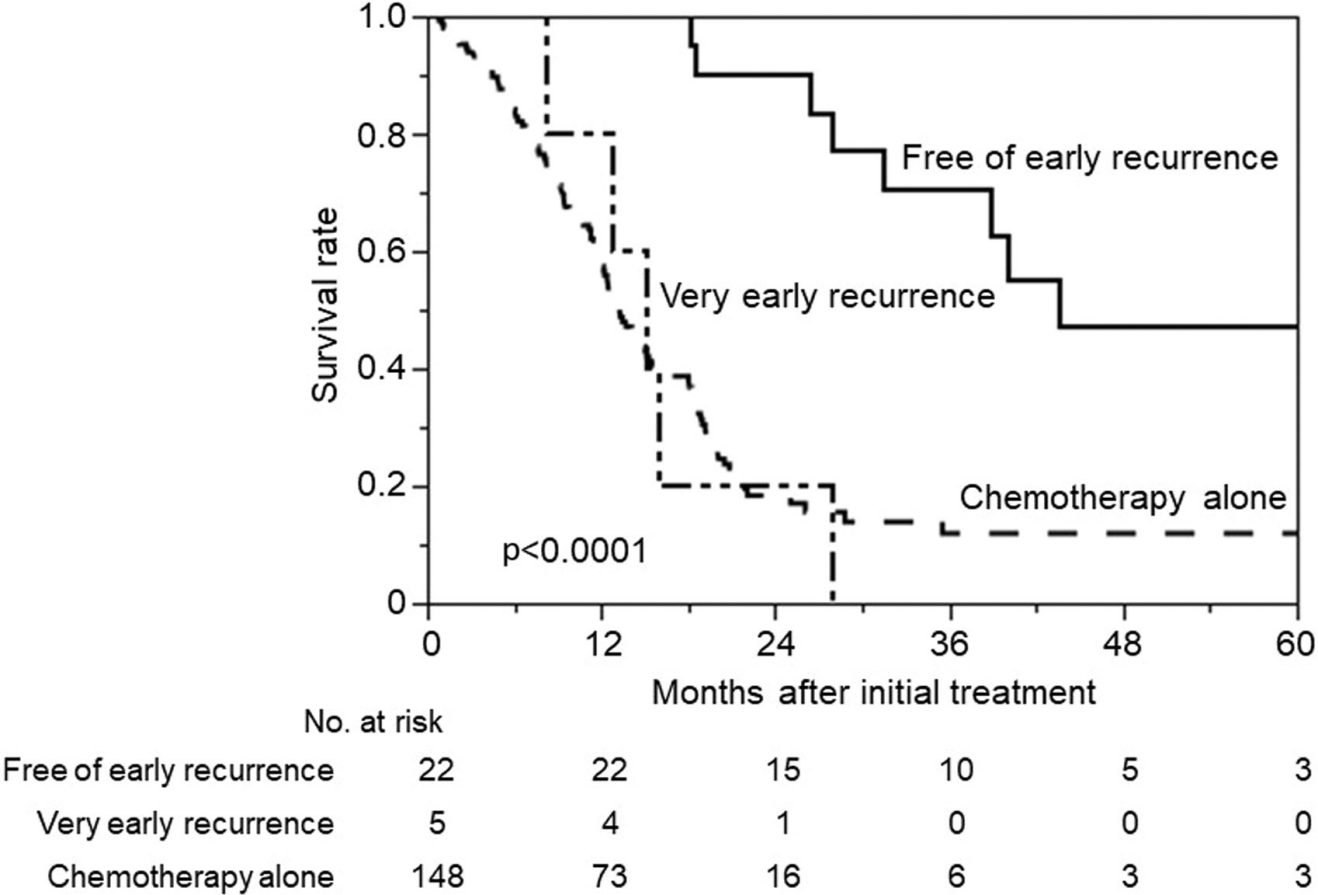
Kaplan–Meier analysis of overall survival in the free of early recurrence, very early recurrence, and chemotherapy alone groups after initial treatment.

MSTs in the VER and FER groups were 15.2 and 44.1 months, respectively (Figure 2). The VER group had a significantly worse survival after initial treatment (*p* < 0.0001). Survival in the VER group was similar to that of the chemotherapy alone group.

## 4 DISCUSSION

This study demonstrated that CS is an effective treatment for stage IV GC but VER after CS is associated with poor prognosis. We found that preoperative PNI <40 and liver metastases were associated with VER after CS. To the best of our knowledge, this study was the first to investigate the adverse effect of VER after CS.

Consistent with previous studies,^5^, ^6^, ^7^ we showed that patients with advanced GC who underwent CS had longer survival than those who underwent chemotherapy alone. Therefore, we conclude that CS is an effective treatment for advanced GC in patients who can undergo surgery with curative intent. However, its survival benefit seems to be limited in patients with VER within 6 months. CS may contribute to poor prognosis in patients with VER; therefore, identification of risk factors for VER after CS may help avoid unnecessary surgery.

In our study, there were more patients with preoperative PNI <40 and with anastomotic leakage in the VER group. Previous studies showed that low PNI was associated with postoperative complications, mortality, and poor prognosis.^17^, ^18^, ^19^ On the basis of these results, low PNI may lead to postoperative adverse outcomes such as anastomotic leakage, early recurrence, and poor prognosis in CS patients. We suggest that decreased compliance with chemotherapy leads to worse prognosis because postoperative adverse outcomes may delay initiation and decrease tolerability of chemotherapy. The European Society of Clinical Nutrition and Metabolism Guidelines on clinical nutrition in surgery recommend nutritional therapy prior to major surgery in patients with severe nutritional risk, even if neoplastic surgery needs to be delayed.^20^ It is important to maintain nutritional status during chemotherapy and preoperatively for advanced GC patients. CS may be avoided in some patients who have poor nutritional status despite nutritional therapy during chemotherapy. PNI for advanced GC patients may be useful for making a decision about CS because it can be easily assessed during chemotherapy.

CS may not be suitable for some patients who have liver metastasis before initial treatment. Tsurumaru et al. reported that tiny lesions or microscopic metastases are not detectable even with the use of high‐resolution imaging.^21^ Shirasu et al. reported that the indications for hepatectomy for multiple liver metastases remain unclear.^22^ We also found that liver metastases were associated with VER. Based on these results, CS for patients with liver metastasis at the time of initial treatment should be carefully considered.

Our study had several limitations. First, it was a retrospective study performed at a single institution and the sample size was small. Therefore, we could not study patients according to the popular four‐category criteria previously published.^23^ Second, the period in which the patients were treated extended over a decade and the surgical procedure and chemotherapy may have changed. Therefore, further work with a prospective cohort study in multiple institutions is warranted to clarify the indications for CS for stage IV GC. Third, we were not able to compare the quality of life between the VER group and the chemotherapy alone group. If the quality of life can be maintained even in the VER group, CS may be considered as an option. Despite these limitations, PNI is useful for making decision about CS because it can be easily assessed in clinical practice.

In conclusion, this study was the first to investigate the adverse effect of VER after CS for stage IV GC. We need to carefully consider the indications for CS, especially for patients who have poor nutritional status and liver metastases. We believe that maintenance of nutritional status during chemotherapy is important. Consideration of PNI when making a decision about CS may avoid unnecessary surgery and improve prognosis.

## AUTHOR CONTRIBUTIONS

All authors contributed to the study conception and design. Material preparation, data collection, and analysis were performed by Atsushi Morito, Kojiro Eto, Masaaki Iwatsuki, Tasuku Toihata, Keisuke Kosumi, Shiro Iwagami, Naoya Yoshida, and Hideo Baba. The first draft of the manuscript was written by Atsushi Morito and all authors commented on previous versions of the manuscript. All authors read and approved the final manuscript.

## FUNDING INFORMATION

This study did not receive funding.

## CONFLICT OF INTEREST STATEMENT

Hideo Baba is an editorial board member of *Annals of Gastroenterological Surgery*. None of the other authors have any conflicts of interest or financial ties to disclose.

## ETHICS STATEMENT

Approval of the research protocol: The protocol for this research was approved by a suitably constituted Ethics Committee of the institution and it conformed to the provisions of the Declaration of Helsinki. Ethics Committee for Epidemiological and General Research at the Faculty (Approval No. 1037).

Informed Consent: N/A.

Registry and the Registration No. of the study/trial: N/A.

Animal Studies: N/A.
